# Understanding Food Insecurity in Westminster, UK: A Community‐Informed Systems Thinking Approach

**DOI:** 10.1111/hex.70914

**Published:** 2026-10-09

**Authors:** Charan Bijlani, Jessica Ellis, Birgit Kopainsky, Cornelia Junghans, Eszter P. Vamos, Paraskevi Seferidi

**Affiliations:** ^1^ Public Health Policy Evaluation Unit, School of Public Health Imperial College London London UK; ^2^ Community Health and Wellbeing Worker Team Paddington Development Trust London UK; ^3^ System Dynamics Group University of Bergen Bergen Norway; ^4^ Evaluation and Innovation, North West London Applied Research Collaboration Imperial College London London UK

**Keywords:** causal loop diagram, community‐based system dynamics, food insecurity, group model building, health inequalities, systems‐thinking

## Abstract

**Introduction:**

Food insecurity is increasing in the United Kingdom and is a growing public health concern. In Westminster, a highly diverse London borough, rates continue to rise in the most deprived areas, such as the Church Street ward, despite increasing food support and local policies.

**Methods:**

This study used a community‐informed systems thinking approach to understand the local dynamics. 26 stakeholders from 6 local services participated in group model‐building (GMB) workshops and interviews to provide insight into how services and policies interact with the structural drivers of food insecurity.

**Results:**

We developed six causal loop diagrams (CLDs) to map interactions between support services, the food environment, local policies, and the drivers of food insecurity. Four system archetypes were identified: Fixes that Fail, Shifting the Burden, Limits to Success, and Success to the Successful, which explain how well‐intentioned policies and interventions can produce unintended consequences that reinforce food insecurity. While identified in Church Street, these archetypes reflect recurring patterns of behaviour that may operate in other highly deprived and ethnically diverse urban settings. The CLDs show how charities, support services, and local communities work together to support residents, but are constrained by short‐term fixes, limited funding, competing priorities, and the local food environment. This results in interventions that focus on addressing immediate needs, rather than the structural drivers such as poverty. Through our evaluation of the GMB approach, participants reported that the workshops were an effective way to share experiences and improve their understanding of food insecurity.

**Conclusion:**

By addressing the feedback mechanisms driving food insecurity and using systems archetypes to identify appropriate solutions, this research provides insights and policy recommendations to create more effective policies across the UK.

**Patient or Public Contribution:**

This article is part of a wider PhD project which involves close engagement and collaboration with public partners. For this article, public partner Jessica Ellis contributed extensively to the design and delivery of the workshops used in this research. She provided support in planning the workshop, designing the evaluation survey, developing workshop materials, co‐facilitating a workshop, interpreting the results, and making contributions to the final manuscript.

## 1 Introduction

The United Kingdom has one of the highest levels of food insecurity in Europe, with an estimated 14% of households experiencing moderate to severe food insecurity [1, 2, 3, 4]. Food insecurity is defined as the ‘inability to reliably afford and access sufficient healthy and nutritious food' [1]. The issue is further exacerbated by the current cost‐of‐living crisis and rising food costs [2]. In the UK, more nutritious and healthy foods cost, on average, three times more per calorie than foods high in sugar, salt, and fat, which pushes households towards cheaper, more processed options [3]. The Food Foundation's ‘Broken Plate 2025' report indicated that the poorest fifth of households would need to spend almost half of their disposable income to meet the government's Eatwell Guide, increasing to 70% for households with children, compared to the 10%–12% for the richest fifth [3, 4]. At the individual level, food insecurity can result in stress and micronutrient deficiencies, and is associated with obesity, hunger, and long‐term mental and physical health conditions, including depression, cardiovascular disease, and type 2 diabetes [1, 3, 5, 6]. Beyond its impact on individual health, poor diet and its related problems are leading risk factors with the greatest impact on the NHS budget. Additionally, food insecurity has been highlighted as a national priority in both the ‘Fit for Future 10 Year Health Plan' and ‘UK Government Food Strategy', demonstrating its importance as a public health issue [3, 7, 8, 9].

Previous research has established key socioeconomic and demographic factors of food insecurity [3, 10, 11], explored the lived experiences of food‐insecure households and the limitations of food aid and local support [12, 13, 14, 15], and evaluated the numerous welfare and food aid policies which aim to manage food insecurity [6, 10, 16, 17, 18, 19, 20, 21]. However, these areas have largely been explored separately, providing a limited understanding of how these factors interact [22]. One way to explore how factors interact is by using a systems thinking approach. This methodology maps the interconnections between key drivers of a problem to understand how their interactions result in outcomes that change over time [23]. Systems thinking has been applied to complex public health problems, where multiple interacting factors can generate unintended consequences [23]. There is also growing interest among local authorities in the UK in applying systems approaches to better understand and respond to public health problems [24]. Recent studies using systems thinking approaches have started to explore some of these interactions in deprived and ethnically diverse communities by examining barriers to healthy eating and community food aid support [25, 26]. While these studies provide important insight into interactions within local food environments and community food support, less is known about how local policies interact with the wider drivers of food insecurity, and how these interactions may affect their intended impact. To better understand these interactions in a local context, it is important to involve stakeholders embedded within the system, as their perspectives can help identify the local mechanisms that maintain food insecurity and inform more effective interventions. Community‐informed systems thinking provides a way to do this by involving local stakeholders most involved in building a shared understanding of the problem [27, 28, 29].

The City of Westminster is a borough in London with extreme health inequalities, where 11% of the population live in areas at the highest risk for food insecurity [30]. Westminster City Council (WCC) has identified food insecurity as a rising issue amongst its residents, with rates of food bank usage increasing three‐fold between December 2021 and May 2022 [31]. Approximately 14% of all residents in the borough live in deprivation, with 23% of all children in the borough affected. Additionally, Westminster is a highly deprived and ethnically diverse borough, where residents experience overlapping socioeconomic and health‐related conditions that increase their risk of food insecurity. Over a third of Westminster residents were born outside of the UK [32], and almost half of residents identify as Black, Asian, or other non‐white ethnic background [33]. WCC and The Food Foundation have identified that individuals from minority ethnic groups experience poorer health outcomes and are at an increased risk of food insecurity compared to those from white ethnic groups [3, 33, 34, 35].

Westminster provides an important setting to explore how local responses interact with the wider drivers of food insecurity. High levels of deprivation and ethnic diversity place residents at an increased risk of food insecurity, which has prompted an extensive network of council and voluntary‐sector responses. In Westminster, these responses provide both immediate food access and support for underlying determinants of food insecurity, such as poverty, healthcare access, and employment. Yet, despite food aid being available 6 days a week across the borough [36], numerous organisations providing wraparound services, and additional council funding dedicated to reducing food insecurity, rates continue to rise [37, 38]. Therefore, this study aimed to investigate how local policies, services, and drivers interact to perpetuate food insecurity in Church Street, Westminster, using a community‐informed systems thinking approach.

## 2 Materials and Methods

### 2.1 Community‐Informed Systems Thinking

This study used a community‐informed systems thinking approach engaging with stakeholders through group model building (GMB) workshops. GMB workshops use structured exercises to share insights and create visual maps known as causal loop diagrams (CLD) [28, 39, 40]. CLDs are visual representations of interconnections that form feedback loops. The CLDs created illustrate how food insecurity and local services interact in Westminster [41].

### 2.2 Setting

This research primarily focused on the ward of Church Street in northwest Westminster, London, UK and its surrounding areas. Wards are small local areas defined by geographical location and used for electoral purposes. The Church Street ward of Westminster falls within the 10% most deprived nationally and has the highest rate of ethnic diversity in the borough [30, 33, 35]. These intersecting characteristics of deprivation and ethnic diversity contribute to the heightened vulnerability of Church Street residents to food insecurity.

### 2.3 Participants and Recruitment

In line with the community‐informed systems thinking approach and policy focus of this research, stakeholders were identified as employees at key services in Westminster who had a role related to food insecurity, supported food‐insecure residents, and had direct knowledge of local policies and services. Several stakeholders were also residents in Church Street and the surrounding areas. This recruitment approach was also informed by recommendations that potentially vulnerable groups should be involved in research only where the research question cannot be addressed through engagement of less vulnerable participants [42].

Services were identified and recruited through purposive sampling by leveraging existing networks, and included health workers, local food aid services, charity support services, and local Citizens' Advice services. Ten different organisations were contacted via email and recruited between February‐May 2025. No participants dropped out of the workshops. Participants provided written informed consent and were reimbursed for their time.

Ethical approval for the workshops was granted by Imperial College London Research and Governance Integrity Team (ref: 7437722).

### 2.4 Design

Between April and July 2025, a total of six sessions were conducted using a GMB approach, including two in‐person workshops (*n* = 11 and 10) and four online interviews (*n* = 1‐2 per interview). Given the study aim of understanding how local services and policies interact with food insecurity, we defined ‘community' in this study as service providers. We identified services through an established Westminster City Council network of local services which aim to address food poverty in the area and invited representatives from several services. A total of 26 participants from 6 different services attended. The number of participants and workshops/interviews were guided by GMB methods, which focus on including stakeholders with relevant knowledge rather than achieving a representative sample or traditional qualitative data saturation [27]. In‐person workshops were held in familiar community spaces and online interviews were conducted via Microsoft Teams. Workshops and interviews ranged from 1 to 2.5 h and were recorded with participant consent. CB designed and facilitated all workshops and interviews. JE (community health and wellbeing worker) supported the design and facilitation of the first workshop with community health and wellbeing workers. All workshops and interviews followed a similar GMB approach using structured exercises, documented as scripts [40]. Participants were first introduced to the session and key system dynamics concepts. They then participated in a ‘Variable Elicitation' exercise, during which participants identified potential drivers and outcomes of food insecurity (workshops 1 and 2), or how their service supports residents experiencing food insecurity (interviews 1–4). The next activity aimed to elicit information on how variables are interconnected either through a ‘Connection Circles' exercise, where participants placed variables around a circle and used arrows to show how they were connected, or a ‘Causal Loop Diagramming', where participants directly mapped connections between variables. Participants illustrated the interconnections between variables and emerging feedback loops in small groups and then shared the rationale with the wider group. Where different perspectives arose, these were discussed as a group, and variables or connections were refined following the discussion. This aimed to identify interconnections between the variables identified in the first activity and highlight feedback loops that drive behaviours in the local environment. The facilitation structure was adapted from similar studies [43] (See Table 1 for workshop details).

**Table 1:** Description and format of workshops.

	Workshop 1	Workshop 2	Interviews 1–4
Format	In‐person, 2.5 h	In‐person, 2 h	Online, 1 h
Facilitators	JE, CB	CB	CB
Number of participants	11	10	1 or 2 per interview
Activity 1	Introduction	Introduction	Introduction
Activity 1 Description	Presentation to set out aims for the workshop, define food insecurity, and introduce participants to systems thinking concepts		
Activity 2	Variable Elicitation	Variable Elicitation	Variable Elicitation
Activity 2 Description	Participants were prompted to identify the key drivers and outcomes of food insecurity in Church Street/surrounding areas and individually write down as many variables as they can think of on different post‐it notes. Every participant then shared one of their variables out loud with the rest of the group	Participants were prompted to identify the key drivers and outcomes of food insecurity in Church Street/surrounding areas and individually write down as many variables they can think of on different post‐it notes. Every participant then shared one of their variables out loud with the rest of the group	Participants were prompted to identify how the service the work for helps food insecure residents in Church Street or surrounding areas, variables were shared out loud with the facilitator who made note of the variables
Activity 2 output	List of variables on post‐it notes	List of variables on post‐it notes	List of variables online
Activity 3	Connection Circles	Creating causal loop diagram from variable list	Causal loop diagramming in small groups
Activity 3 Description	Participants were arranged into smaller groups and made connections between variables identified in the variable elicitation activity by drawing arrows to indicate how they are connected	This activity was conducted in parallel to Activity 2. As participants shared variables, CB drew individual feedback loops for participants to see	Participants were guided by the facilitator to make connections between variables identified in the variable elicitation script using Kumu. io software with formed CLDs
Activity 3 output	Four connection circles were developed, showing the variables and causal connections used to develop the CLDs	One CLD was developed collaboratively with participants	One CLD was developed collaboratively with participants during each interview

### 2.5 CLD Synthesis

Following each GMB workshop or interview, we reproduced the co‐developed CLDs in Kumu. io, with the exception of Workshop 1, for which the research team developed the CLD informed by co‐developed Connection Circles. We then reviewed variable lists alongside audio recordings and transcripts to identify and incorporate additional variables into each CLD. Where needed, we consolidated duplicate variables and variables with different names, where participants' descriptions indicated that they represented similar concepts. This iterative process resulted in a CLD for each workshop or interview. The individual diagrams were synthesised into an overarching CLD. The first workshop CLD was reviewed by our community partner (JE), and all CLDs were reviewed by the research team. CLDs consist of feedback loops, which can either be balancing or reinforcing. Balancing feedback loops are labelled using a ‘B' and are called balancing because they stabilise the system and counteract changes. Reinforcing feedback loops are labelled with an ‘R' and indicate a change that is amplified in the same direction as the initial change [41]. During the synthesis process we aimed to identify feedback loops, and through this, patterns emerged that reflected established systems archetypes [44]. Systems archetypes are recurring patterns of behaviour that can reveal system structures that drive a problem [44] and help explain why well‐intentioned policies can sometimes lead to unintended consequences [45]. The first archetype emerged naturally from the synthesis process, which resulted in using system archetypes as a conceptual tool to identify and analyse other recurring patterns in the CLD [46]. The overarching CLD was then organised into six final CLDs, each representing a distinct theme or archetype identified during the synthesis process. The final CLDs map key interconnections and feedback structures that drive food insecurity in Westminster.

### 2.6 Evaluation

Participants were asked to anonymously complete online pre‐and post‐questionnaires (questionnaires can be found in Appendix 1) to assess changes in their mental models and to gather feedback on the group model building process, following a similar format used in previous GMB evaluations [47]. To evaluate the change in mental models, the pre‐questionnaire included two open‐ended questions which asked participants to identify factors and actions related to food insecurity. These same questions were asked again in the post‐questionnaire to measure change. To evaluate the process of the workshop, eight post‐questionnaire survey questions were developed using the ‘Comprehensive multi‐scale evaluation framework for Participatory Modelling programmes’ [48]. These included 5 Likert scale questions, which ranged from ‘not at all' to ‘extremely', 3 open‐ended questions, and one final question, that allowed participants to include any additional comments or feedback. Survey results were analysed by coding open‐ended responses into themes and calculating descriptive statistics for the Likert‐scale questions.

## 3 Results

### 3.1 CLD Overview

Six synthesised CLDs were created to illustrate the key dynamics contributing to food insecurity in Church Street and the surrounding areas in Westminster. Each CLD focuses on a different theme that emerged from the workshops and five CLDs represent specific systems archetypes (see Table 2 for a description of the system archetypes identified).

**Table 2:** Description of four systems archetypes.

Archetype	Description	Resulting behaviour pattern	Solution to the archetype
Fixes that Fail	This archetype highlights a dynamic where a quick solution (‘fix’) is applied to solve a problem. Initially, the solution improves through a balancing loop, which stabilises the issue in the short‐term. Over time however, reliance on the ‘fix’ results in unintended consequences which can worsen the original problem and create a reinforcing feedback loop. The defining feature is that the unintended consequence is generated by the ‘fix’ itself [44]	Initial improvement from the ‘fix’ is followed by worsening due to the implemented ‘fix’ which causes unintended consequences.	Reduce or avoid the unintended consequence generated by the fix and implement longer‐term responses to the underlying problem.
Shifting the Burden	This archetype emerges when attention is directed towards one type of solution, which may be easier or more achievable, opposed to addressing the root cause of the problem. A balancing loop provides temporary relief from the problem however, continued reliance on the short‐term solution diverts attention and capacity away from the fundamental solution [44].	The problem continues to be managed in the short term while reliance on the symptomatic solution increases and the fundamental response becomes weaker or deprioritised.	Maintain necessary short‐term support while strengthening the fundamental response and reducing dependence on the symptomatic solution.
Limits to Success	In this archetype, an intervention or ‘initial effort’ initially leads to an improvement through a reinforcing loop. However, progress eventually slows down because of a ‘limiting factor' that constrains the continued success of the intervention and is not addressed. This results in a balancing loop that limits further improvement, rather than the intervention itself worsening the initial problem [44]	Initial improvement slows as the limiting factor constrains success	Identify and address the limiting factor that constrains the continued success of the intervention.
Success to the Successful	This archetype describes how allocating more resources to one solution increases its chance of being successful compared to other solutions. The initial success results in a reinforcing loop which continues to direct more support to the same solution, supporting its continuous success. However, this concentration of resources results in less support for other solutions, which are unable to succeed, even if they might have better potential for success [44]	One response becomes successful and well‐resourced, while competing responses become under‐resourced and less able to demonstrate success.	Change how resources are allocated and success is assessed so that alternative responses have an opportunity to demonstrate their value

### 3.2 Corporate Social Responsibility CLD

The Corporate Social Responsibility CLD (Figure 1) illustrates how the redistribution of surplus food from corporations to the charity sector addresses immediate food insecurity yet places unintentional strain on the charities receiving the donations. Food donations from corporations can improve immediate access to food by supplying local food banks, which also helps reduce food waste (Figure 1 loop B1). However, as donations increase, demand is placed on charities to collect, store, and redistribute the food effectively. This logistical burden creates a reinforcing loop (Figure 1 loop R1), further stretching the capacity of already resource‐limited, volunteer‐operated services and undermining the intended effect of reducing food insecurity. This intervention‐generated consequence demonstrates a Fixes that Fail' systems archetype [44], where a short‐term solution, like surplus food donation (the ‘fix’), temporarily addresses the symptom of food insecurity, but generates an unintended consequence by burdening charities [46]. One workshop participant highlighted that “*(charities) have to manage the logistics of storing (food) and then delivering it to a now wider and wider network… So while supermarkets who are culpable for all the producing, they are actually rewarded for giving it out, and charities have to, in a way, pay the price*”.

**Figure 1:**
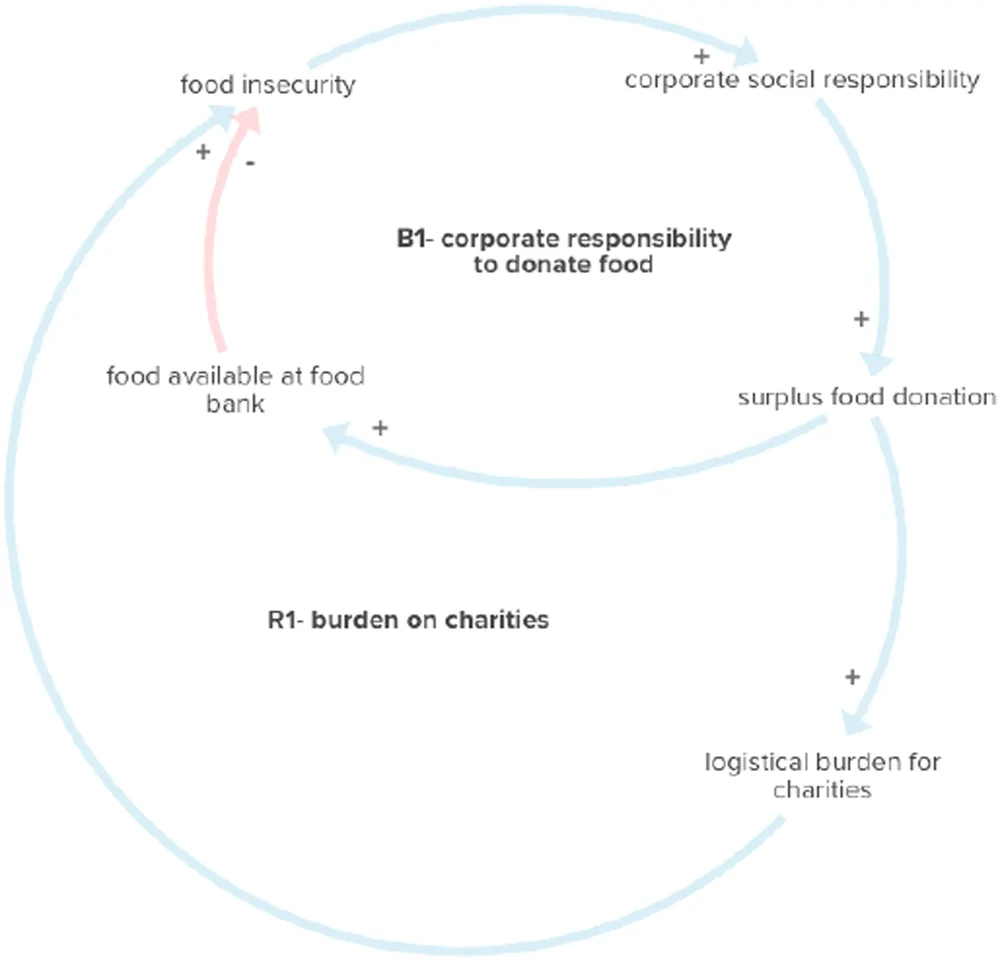
Corporate social responsibility CLD. Synthesised causal loop diagram of how surplus food is redistributed. Red arrows indicated negative (–) polarity. Blue arrows indicated positive (+) polarity. B1 indicates a balancing loop. R1 indicates a reinforcing loop. Additional information about this CLD is publicly available at: https://kumu.io/cbijlani/understanding-food-insecurity-in-westminster-uk-a-community-informed-systems-thinking-approach#figure-1.

### 3.3 Support Services and Poverty CLD

The Support Services and Poverty CLD (Figure 2) describes how short‐term support services are used to manage food insecurity, alongside the barriers that limit access to them. In Westminster, support services such as food banks, pantries, community wellbeing workers, and Citizens Advice, offer a range of support, including benefit advice, debt advice, cooking support, employment guidance, and more. These services can help decrease food insecurity in the short term by addressing immediate needs (Figure 2 Loop B1). However, participants described several barriers that limit residents' ability to engage with these services. These included stigma, cultural and religious norms, and mental health challenges. One participant noted that they have to encourage residents to continue to seek support during the month of Ramadan, reminding them that they are *‘zakat worthy'*, meaning they should still access charity support and that *“it's not a bad thing that you're doing (seeking support)”*. Mental health was a barrier raised across all six workshops and interviews. Participants noted that poor mental health can reduce an individual's ability to access services, and that cultural understandings of mental health influenced whether residents felt able to discuss their needs (Figure 2 Loop B3).

**Figure 2:**
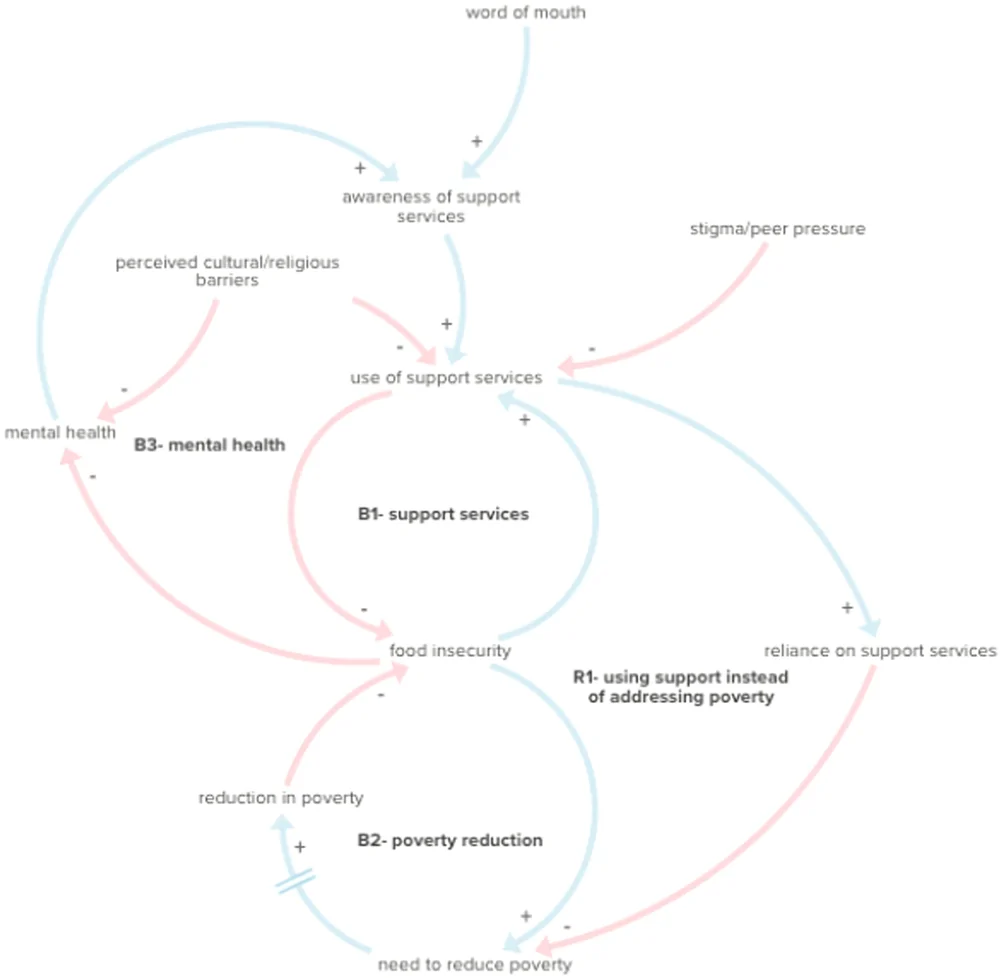
Support services and poverty CLD. Synthesised causal loop diagram of food insecurity support services and barriers to access. Red arrows indicate negative (–) polarity. Blue arrows indicate positive (+) polarity. B1–B3 indicate balancing loops. R1 indicates a reinforcing feedback loop. Arrows that are intersected by parallel lines indicate a ‘time delay’, where the effect of one variable on another does not occur immediately. Additional information about this CLD is publicly available at: https://kumu.io/cbijlani/understanding-food-insecurity-in-westminster-uk-a-community-informed-systems-thinking-approach#figure-2.

Although these barriers limit access to support services, improving service uptake alone will not address the underlying causes of food insecurity. Figure 2 highlights that addressing these barriers may improve food insecurity in the short term, but deeper structural drivers such as poverty will remain unaddressed. Participants described that continuous use of support services creates a reinforcing loop (Figure 2 Loop R1), where individuals and systems rely on these services as the default way of managing food insecurity. As one participant noted: *“it's impossible to get out (of needing support services). Yeah, it's impossible unless you win the lottery or get a fantastic job. And most (people) are not in that position”*. This dynamic reflects a ‘Shifting the Burden' systems archetype, where short‐term responses are prioritised to manage symptoms, while longer‐term, more fundamental solutions are seen as difficult and time‐consuming and are therefore deprioritised (Figure 2 Loop B2). Over time, a growing reliance on support services reduces the attention and capacity given to poverty reduction, reinforcing the system's dependence on short‐term support services (Figure 2 Loop R1).

### 3.4 Voucher Limits CLD

The Voucher Limits CLD (Figure 3) illustrates how supermarket vouchers provided by support services in Westminster are initially effective in reducing food insecurity but have limitations. Supermarket vouchers offer food support by allowing residents to purchase culturally appropriate food that meets their dietary needs. Initially, as more vouchers are administered, food insecurity decreases (Figure 3 Loop R1). As rates of food insecurity decrease, it is assumed that the vouchers are a successful policy because they appear to solve the problem. However, as one participant noted: *“even the food voucher is a Band‐Aid…it's just a stop gap arrangement, so that you don't starve today. Tomorrow again you're back because, well, you don't have enough”*. While vouchers offer immediate food support, their success cannot be sustained. Referral organisations have monthly quotas that limit the number of vouchers they can administer, and each household is only eligible for a limited number of vouchers (Figure 3, Loop B1). This demonstrates a ‘Limits to Success' systems archetype [44], where vouchers initially lead to an improvement, so they are further prioritised as a solution, but referral quotas and limits that accompany them constrain their continued use. As one participant who refers residents for vouchers noted, “*Even with the vouchers, the number of people we can refer is so limited right now…I was looking at somebody's notes today and thought ‘oh my god, you poor thing'. She should have got one, but I finished my quota”*. Another participant noted the difficulty in the voucher limits and quotas, stating, *“it's quite heartbreaking. It's like during COVID. Remember they had to decide who lives, who dies. It's not as bad as that. I mean, our decisions are not so life impacting, but yes it is. I wish we had enough for all the people who needed it”*.

**Figure 3:**
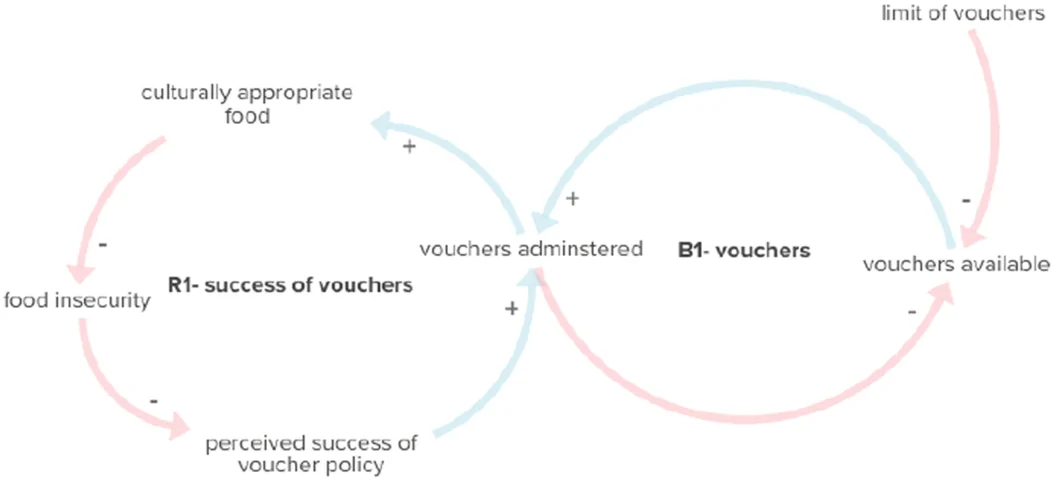
Voucher limits CLD. Caption: Synthesised causal loop diagram of food insecurity support services and barriers to access. Red arrows indicate negative (−) polarity. Blue arrows indicate positive (+) polarity. B1 indicates a balancing loop. R1 indicates a reinforcing feedback loop. Additional information about this CLD is publicly available at: https://kumu.io/cbijlani/understanding-food-insecurity-in-westminster-uk-a-community-informed-systems-thinking-approach#figure-3.

### 3.5 Reliance on Community Support CLD

The Reliance on Community Support CLD (Figure 4) illustrates how short‐term reductions in food insecurity are achieved through community support, which over time becomes the expected response. This dynamic results in more fundamental, long‐term solutions, such as improved government policies, being deprioritised. As food insecurity increases, community support often expands in response (Figure 4 loop B1). Participants described how community groups, religious organisations, and volunteers step in to provide informal food aid or culturally appropriate meals. While these efforts help to reduce food insecurity initially, they also reinforce expectations that communities will continue to fill these gaps (Figure 4 Loop R1), thereby reducing pressure on national or local governments to implement more effective policy responses (Figure 4 Loop B2). One example discussed by participants was a policy that temporarily housed refugees and asylum seekers in hotels across Westminster. This policy failed to consider the dietary and cultural food needs of the population, which resulted in food insecurity and a rise in food bank referrals. In response, participants noted that the local community stepped in to provide appropriate food, “*we sort of (asked) the local church and local charities to contribute a bit more for the communities. And then I think what, like even one of the mosques they made, like homemade food, we had an Afghani imam that was like, OK, they eat these types of foods. Like I'm happy to make and bring some of this around”*. This reflects a ‘Shifting the Burden' systems archetype [44], where reliance on community support acts as a temporary solution, but ultimately deflects responsibility away from much‐needed policy changes, which are often more resource‐intensive and take time to implement, thereby reinforcing community support as the expected response.

**Figure 4:**
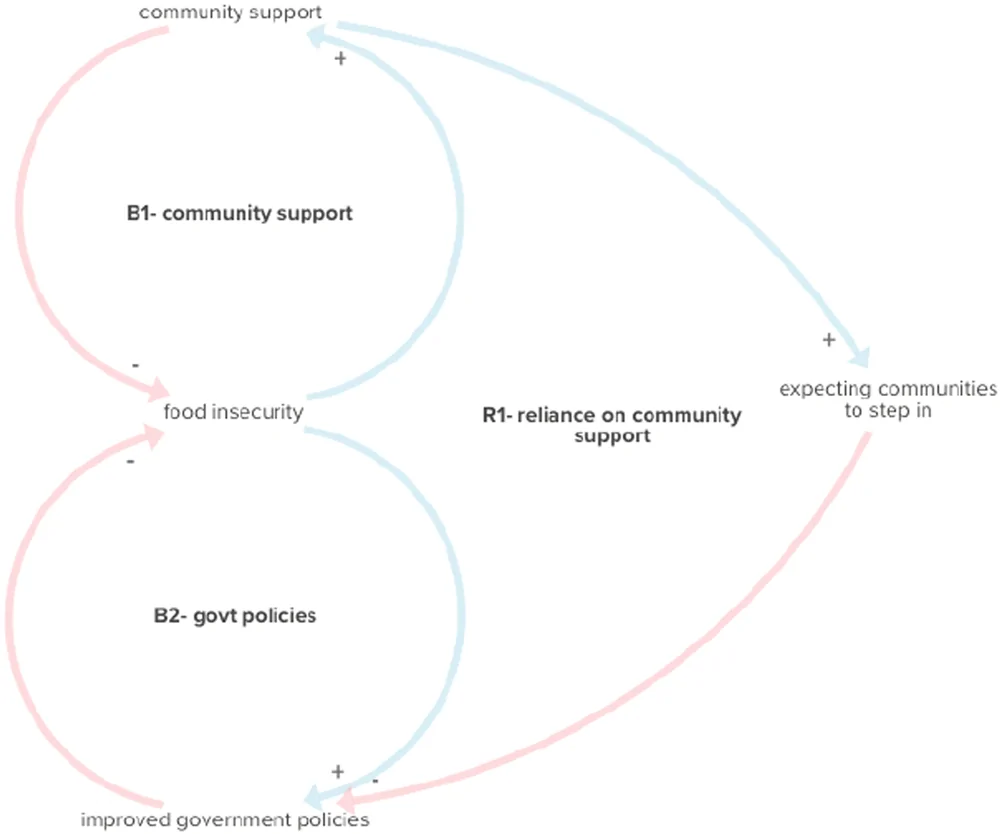
Reliance on community support CLD. Synthesised causal loop diagram of food insecurity support services and barriers to access. Red arrows indicate negative (−) polarity. Blue arrows indicate positive (+) polarity. B1 and B2 indicate balancing loops. R1 indicates a reinforcing feedback loop. Additional information about this CLD is publicly available at: https://kumu.io/cbijlani/understanding-food-insecurity-in-westminster-uk-a-community-informed-systems-thinking-approach#figure-4.

### 3.6 Funding Competition CLD

The Funding Competition CLD (Figure 5) highlights how limited funding is often directed towards managing hunger, a symptom of food insecurity, at the expense of other potentially effective solutions. As food insecurity increases, the first visible symptom is often hunger, which results in funding being directed towards food aid services that provide immediate relief, such as food banks and pantries (Figure 5 loop R1). Food aid services successfully help reduce hunger in the short term, which increases their perceived effectiveness and reinforces further funding from local or national governments. Simultaneously, services that provide individualised support struggle to receive funding. These services include support such as one‐to‐one benefit advice, debt support, skill building, or wraparound care, which aim to reduce food insecurity by addressing its root causes. Without adequate funding, these interventions are unable to demonstrate their success in reducing food insecurity (Figure 5 Loop R2). Participants highlighted the need for more individualised support services, stating “*(the) problems people face are not all the same. Just as no two people are the same”*. The dynamics in Figure 5 reflect a ‘Success to the Successful' systems archetype [44], where one solution becomes dominant not necessarily because it's more effective, but because the initial success of that solution reinforces the decision to allocate more resources to it. In a resource‐constrained environment, this dynamic limits the success of other, potentially more impactful solutions. As one participant stated: *“it's thought through in a very like immediate and reactive way. It's like OK, if there's food insecurity, we're going to have some pantries. And then you're firefighting needs without an actual solution. But it's the repetitiveness of (funding for food aid) that you're not making any positive changes with that. There's so much potential that you have that you could change, but it's (funding) not being invested in the right way … there's been so many mistakes done by so many of the governments…”*.

**Figure 5:**
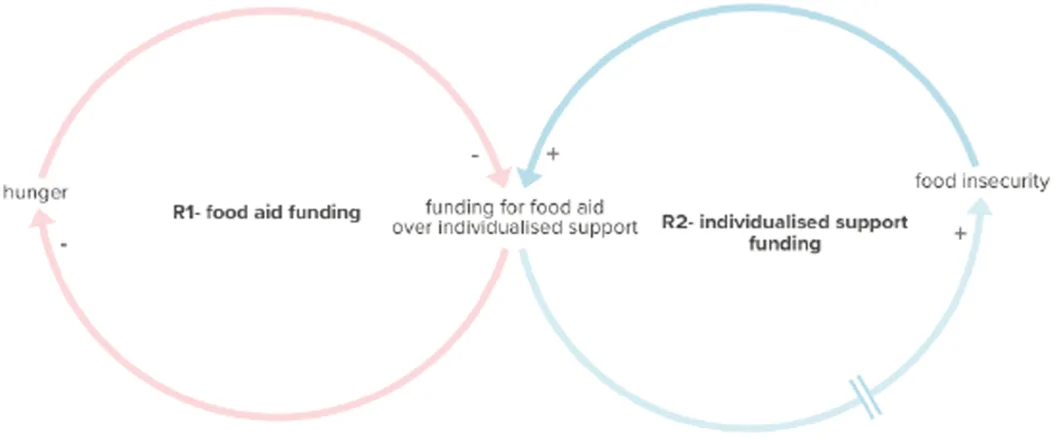
Funding competition CLD. Synthesised causal loop diagram of food insecurity support services and barriers to access. Red arrows indicate negative (−) polarity. Blue arrows indicate positive (+) polarity. R1 and R2 indicate reinforcing feedback loops. Arrows that are intersected by parallel lines indicate a ‘time delay’, where the effect of one variable on another does not occur immediately. Additional information about this CLD is publicly available at: https://kumu.io/cbijlani/understanding-food-insecurity-in-westminster-uk-a-community-informed-systems-thinking-approach#figure-5.

### 3.7 Local Food Environment CLD

The Local Food Environment CLD (Figure 6) illustrates how poor access to good quality and affordable food in the Church Street area reinforces food insecurity. The local food environment in Church Street consists of a large market and numerous takeaway and fast‐food options. Participants noted that there is limited access to affordable retailers in the area, and to access more affordable options, residents must travel to other parts of the borough, which can be time‐consuming and costly (Figure 6 Loop R1). As a result, the local market is favoured by residents because of cultural familiarity and the ability to communicate with vendors in their native language. As one participant noted, *“sometimes, like the community that live there will trust the people that communicates the best with them”*, highlighting how cultural familiarity can shape food choices. Unfortunately, the local market was also described as offering poor‐ quality produce with participants stating: *“many times I buy, I throw it”* and that *“the market is not cheap like we think, and sometimes the market is very expensive”*, highlighting the limitations of the local and culturally appropriate food options (Figure 6 Loop R5). Alongside the market, there is an abundance of takeaways and fast‐food options, which further limits access to healthy food (Figure 6 Loop R2). Residents have no choice but to turn to these options due to a lack of affordable alternatives, reinforcing a reliance on the local food environment, which further contributes to food insecurity.

**Figure 6:**
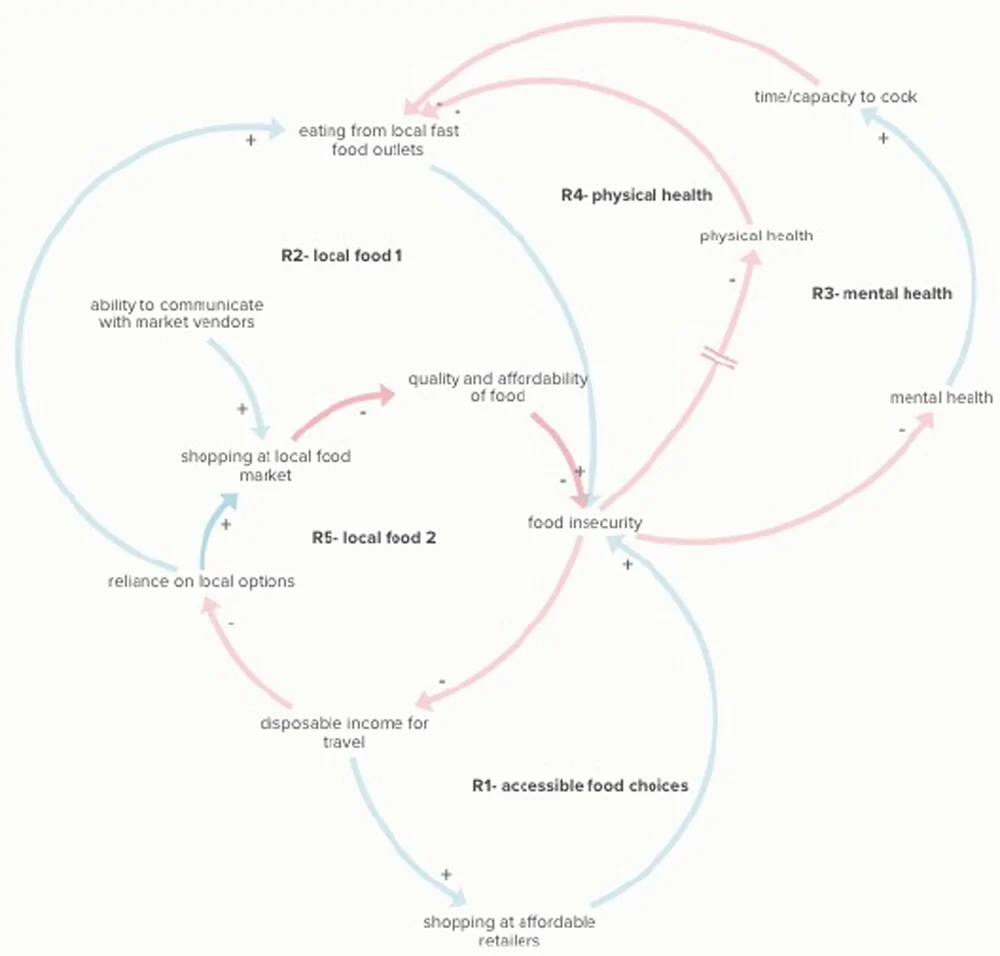
Local food environment CLD. Synthesised causal loop diagram of food insecurity support services and barriers to access. Red arrows indicate negative (−) polarity. Blue arrows indicate positive (+) polarity. R1‐R5 indicate reinforcing feedback loops. Arrows that are intersected by parallel lines indicate a ‘time delay’, where the effect of one variable on another does not occur immediately. Additional information about this CLD is publicly available at: https://kumu.io/cbijlani/understanding-food-insecurity-in-westminster-uk-a-community-informed-systems-thinking-approach#figure-6.

Participants described how closely connected food insecurity is to both physical and mental health (Figure 6 Loops R3 and R4). Food insecurity can worsen health conditions by reducing someone's ability to travel to purchase food, their capacity to cook, or plan meals and more. These health‐related constraints increase eating from local fast‐food outlets, reinforcing food insecurity. Additionally, peer pressure and cooking skills were highlighted by participants as factors that contribute to relying on local fast‐food outlets, perpetuating food insecurity in the local environment.

### 3.8 Survey Results

A total of 18 participants completed both the pre‐ and post‐workshop surveys. The pre‐and post‐surveys included the same two open‐ended questions, which asked participants to identify factors contributing to food insecurity and actions to reduce it.

Responses to these questions were coded into six themes. Responses from the pre‐workshops survey most frequently emphasise financial constraints (e.g. cost of food) and food access. Whilst post‐workshop responses placed greater emphasis on physical/mental health, and skills and knowledge (Table 3). The word cloud (Figure 7) illustrates responses in the pre‐and post‐workshop surveys, with larger words representing factors mentioned more frequently by participants.

**Table 3:** Thematic coding and frequency of factors identified in pre‐ and post‐workshop survey.

Theme	Frequency in pre‐survey	Frequency in post‐survey
Financial/income	10	9
Food access/availability	10	6
Cultural needs	1	0
Skills and knowledge	5	7
Physical/mental health and wellbeing	7	10
Social and wider environmental/structural factors	1	4

**Figure 7:**
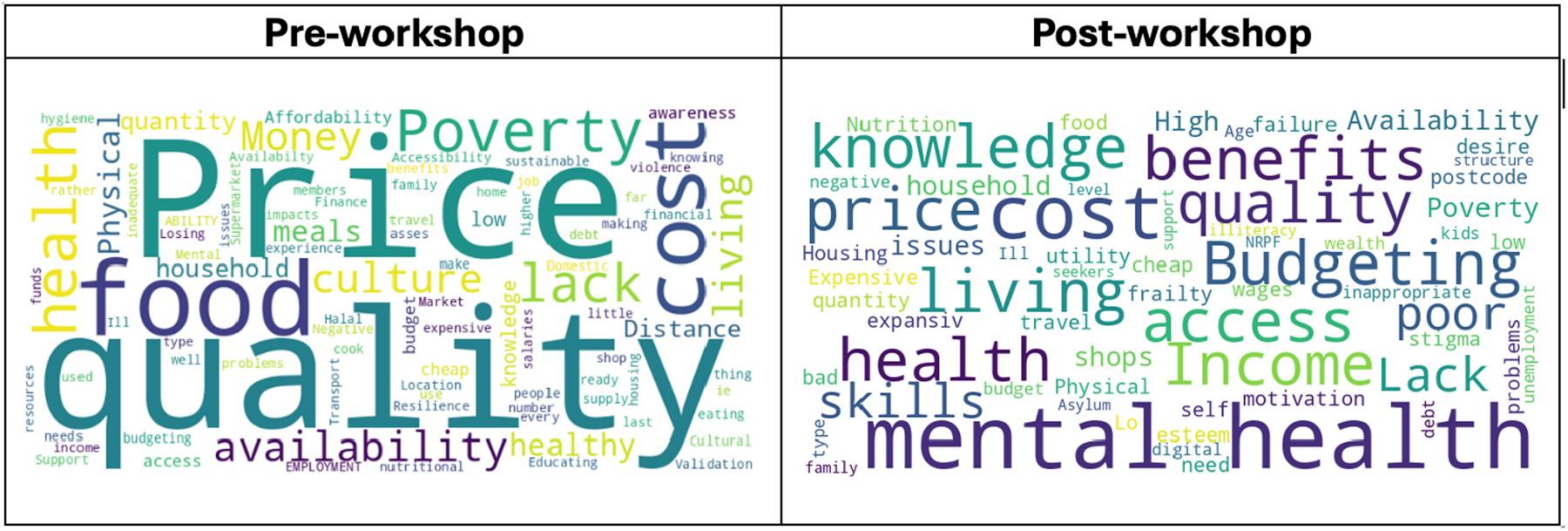
Wordcloud of factors stated in the pre‐ and post‐workshop surveys. Larger works represent factors mentioned more frequently by participants.

Results from the Likert scale (1 = not at all; 5 = extremely) questions indicated high levels of satisfaction with the workshop (Table 4). Participants reported that the workshop was an effective way to share experiences of food insecurity (mean = 4.4, SD = 0.76), and felt comfortable sharing their opinions (mean = 4.7, SD = 0.73). Participants felt that the workshop was relevant to their work (mean = 4.4, SD = 0.83) and that their understanding of food insecurity increased (mean = 4.0, SD = 1.20). Three further open‐ended questions asked participants about their experiences about the workshop, where participants highlighted that they enjoyed seeing *“that most paths lead back to helping the person, not the symptom”*, and hearing others' perspectives.Some participants from the first workshop noted that the ‘Connection Circle' activity had been difficult to understand. Based on this feedback, we adapted the remaining workshops to use an alternative activity (‘Causal Loop Diagramming'), which had the same purpose of connecting variables identified during the Variable Elicitation activity. Overall, participants described the workshop as engaging, interactive and that it provided opportunities to share ideas and feel listened to.

**Table 4:** Likert‐ scale post‐workshop survey results.

Survey question	*N*	Minimum	Maximum	Mean	SD
Did you find the workshop to be an effective way to share how residents in Church Street and surrounding areas are impacted by food insecurity?	18	2	5	4.4	0.76
How comfortable did you feel sharing your opinions during the workshop?	18	2	5	4.7	0.73
Do you feel participating in the workshop was relevant to your work?	18	2	5	4.4	0.83
Do you feel your understanding of food insecurity has improved because of participating in the workshop?	18	1	5	4.0	1.20
Did you gain any knowledge from the workshop that you will be able to apply directly into your work?	18	1	5	3.9	1.10

## 4 Discussion

This study applied a community‐informed systems thinking approach to develop six CLDs that map food insecurity in a highly deprived and ethnically diverse area in London, and the local policies and services that shape it. The diagrams, developed through workshops and interviews with local stakeholders, show how charities, support services, and local communities are working to support residents but face challenges with short‐term interventions, limited funding, competing priorities, and the local food environment. As a result, interventions tend to focus on immediate needs such as hunger, rather than addressing the structural drivers of food insecurity, such as poverty. Survey findings highlight that the GMB workshops effectively encouraged participants to broaden their understanding of food insecurity beyond food access and cost, prompting consideration of other factors such as health, skills and knowledge.

Our findings, which focus on Church Street, Westminster, support those from other ethnically diverse and deprived communities in the UK. Research from other UK cities has shown that limited access to supermarkets leads to greater reliance on fast food outlets [13, 14, 49, 50, 51, 52]. Our study also highlights that reliance on fast‐food outlets is shaped by peer pressure, time scarcity, and social relationships, indicating that takeaway consumption is socially embedded and not just a response to convenience [13]. Religious and cultural norms were also highlighted as barriers to accessing support services in Church Street, similar to findings from Bradford, UK, particularly among South Asian households [49, 50]. Our research additionally shows that these norms further interact with the local food environment by reinforcing reliance on the local market, which is favoured due to cultural familiarity and ease of communication. This dynamic reflects ‘food oppression', where ethnicity, deprivation, and geography intersect to shape unequal food access [53]. These findings reinforce that barriers to food and support are not just logistical (e.g. having access to more supermarkets) but also sociocultural [13].

Consistent with existing research, our study strengthens evidence linking food insecurity to both mental and physical health [13, 15, 54]. Poor health was noted as a factor that limits a person's ability to travel, cook, or access food, resulting in food insecurity, which in turn worsens health outcomes, forming a reinforcing cycle. Previous UK research demonstrates a strong association between food insecurity and long‐standing mental health conditions, with this relationship more pronounced for some ethnic minority groups, where stigma, underdiagnosis, and barriers to service access may further compound risk [55]. By using a systems‐thinking approach, our study offers insights into how cultural and religious barriers influence multiple parts of the system through their interaction with mental health, shaping service uptake, and reliance on local fast‐food outlets, further perpetuating food insecurity.

It is well‐established that food aid, supermarket food donations, and other short‐term support services do not provide long‐term solutions to food insecurity, as they fail to address its root causes, such as poverty [56, 57, 58, 59]. Our research helps to explain why and how these well‐meaning policies implemented across the UK become trapped in cycles that prioritise short‐term fixes and familiar solutions, which often face structural constraints that limit their success. For example, food aid and supermarket donations appear successful because they meet immediate needs and, as a result, become embedded in the system and continue to receive funding. However, by prioritising this policy approach, it limits consideration and investment in other interventions that may be more impactful in the long term. Researchers have argued for a shift toward poverty alleviation and more dignified, cash‐first approaches [58, 60, 61, 62]. Our findings align with these critiques but also demonstrate that when such approaches are capped by referral limits and are not tied to broader long‐term poverty reduction strategies, they end up reinforcing the same short‐term solutions. While previous studies have highlighted how responsibility for addressing food insecurity has shifted to community groups and voluntary services [52, 63, 64], our findings build on this by showing how, over time, the burden of addressing food insecurity becomes normalised as a community responsibility rather than a statutory one. This further reinforces underinvestment in policies that address the structural drivers of poverty. While this study focused on local dynamics, it's important to acknowledge some factors did not emerge during the workshops but are widely documented in the literature. For example, the political pressure to deliver visible, short‐term solutions, combined with constrained local authority budgets can incentivise symptom focused interventions [57]. Local authorities are often tasked with managing national level policy failures, despite lacking formal powers or sufficient resources to address them [65]. The absence of these themes in our work may reflect how structural constraints are experienced differently by local services.

The use of systems archetypes in this study can guide solutions to the problems identified. Firstly, we recommend that redistribution models between supermarkets and charities go beyond the transfer of surplus food, moving towards approaches that weaken the reinforcing feedback loop that adds logistical burden to charities [59]. As supermarkets are rooted in local communities and held to corporate social responsibility standards, they have the capacity to provide more meaningful actions, such as by supporting food price reductions and ensuring employees are paid the Living Wage, alongside implementing longer‐term solutions to food insecurity [59, 66]. We also recommend a reallocation of funding to support longer‐term government interventions that address poverty. This includes allocating ring‐fenced funding for individualised support, particularly mental or physical health services. Because these types of support often take longer to demonstrate impact, protected budgets and evaluation methods that reflect longer‐term benefits could change how success is defined and how resources are allocated, giving more fundamental responses to food insecurity an opportunity to demonstrate their impact. Cash‐first approaches should be expanded; however, this should be accompanied by efforts to identify and address the limiting factors that constrain their reach, such as referral limits [67]. Addressing these limits would allow vouchers to continue to provide short‐term food insecurity relief. In addition, existing short‐term, symptomatic solutions or ‘fixes' such as food aid, vouchers, supermarket donations and support services remain essential and should continue to be delivered alongside structural policies that reduce the long‐term need for such interventions. Finally, these recommendations highlight the need for food insecurity policies that move beyond managing food access or hunger, towards a broader goal of addressing inadequate income and poverty.

This study offers important insights into the dynamics of food insecurity in a highly deprived and diverse ward, with relevance beyond Westminster. A key strength lies in the community‐informed systems thinking approach and working with stakeholders embedded in delivering local services, many of whom are also local residents. This enabled a deeper understanding of the local system through first‐hand insight and a better understanding of the complexity within local communities. The use of systems archetypes to synthesise findings is a novel approach, revealing recurring patterns of policy failure that can be used to identify leverage points and inform more effective and targeted solutions. These patterns are not unique to Westminster with policies such as food aid, support services, and surplus food redistribution implemented widely across the UK. Therefore, the feedback structures and archetypes identified, may be transferable beyond the local context of Church Street facing similar structural constraints and may also be relevant to other complex policy areas beyond food insecurity.

There are limitations worth noting. Although the participants involved represented major food aid services and policy providers, the sample does not capture all perspectives within the local system. The findings are specific to Westminster and may not be directly transferable to other settings, but may still offer insights that can be applied to other multi‐ethnic and highly deprived urban settings. Another limitation worth noting, is typically, participants would be invited to review the causal loop diagrams or the final model. Due to time constraints, reviewing the final model was not possible, and some insights from the workshops may therefore have been missed. The involvement of JE (a local community health worker) throughout the research process helped to mitigate this limitation by providing ongoing feedback. The survey study design and small sample size meant that findings were analysed descriptively and do not allow us to attribute changes in participants' perspectives directly to the GMB process or assess individual‐level changes. However, evaluating the GMB process allowed us to capture changes amongst the group. Although we engaged with local stakeholders, including local residents with direct experience of supporting food‐insecure residents in Church Street, people with lived experience of food insecurity were not included in this study. As a result, the CLDs reflect the perspectives of service providers and other local stakeholders, in line with the study's policy focus, and may not capture all household‐level experiences of food insecurity. Future work should include users of the services, where appropriate, to further strengthen our understanding of how these services impact food‐insecure households.

## 5 Conclusion

This study aimed to understand the local dynamics of food insecurity in Church Street, Westminster, using a community‐informed systems thinking approach. The causal loop diagrams developed through workshops and interviews provided insight into how local services and policies interact with structural drivers such as poverty and the local food environment. By identifying feedback loops and using systems archetypes, the research highlights how well‐meaning food insecurity interventions in Westminster and across the UK can focus on short‐term solutions and result in unintended consequences that cause them to fail. The findings show that in the absence of sustained statutory support, responsibility for managing food insecurity increasingly shifts to voluntary and community services. This shift reinforces funding decisions that favour visible, short‐term responses over more preventative or individualised approaches. Policy recommendations include weakening the reinforcing feedback loop associated with supermarket redistribution, addressing limiting factors such as referral limits that constrain voucher policies, and restructuring funding to prioritise longer‐term, individualised support. Longer‐term support should be provided alongside current short‐term, symptomatic solutions that remain essential, while also moving towards policies that address poverty.

## Author Contributions

**Charan Bijlani:** conceptualisation, investigation, methodology, visualisation, writing – original draft, formal analysis, project administration, data curation, funding acquisition. **Jessica Ellis:** formal analysis, writing – review and editing, investigation. **Birgit Kopainsky:** methodology, writing – review and editing. **Cornelia Junghans:** writing – review and editing, supervision. **Eszter P. Vamos:** writing – review and editing, supervision. **Paraskevi Seferidi:** conceptualisation, methodology, investigation, writing – review and editing, supervision, formal analysis.

## Conflicts of Interest

The authors declare no conflicts of interest.

## Supporting information


Supporting File

## Data Availability

The data that support the findings of this study are available from the corresponding author upon reasonable request.
