# Supplementary material for: Understanding Food Insecurity in Westminster, UK: A Community‐Informed Systems Thinking Approach
Source: Health Expect. 2026 Oct 9;29(5):e70914. doi: 10.1111/hex.70914 (PMC13651544; doi:10.1111/hex.70914)
Supplement: Supplementary file 1 [file HEX-29-e70914-s001.docx]

**Appendix 1**

**Pre-Workshop Survey**

**Post Workshop Survey**
